# Comparing Nutrient Intake by Wolf Spiders (*Hogna carolinensis*) Consuming Frogs (*Acris blanchardi*) and Crickets (*Gryllodes sigillatus*)

**DOI:** 10.1002/ece3.71045

**Published:** 2025-03-02

**Authors:** Chloe G. Hunsucker, Colton Herzog, Jamie T. Reeves, Shawn M. Wilder, Scott T. McMurry

**Affiliations:** ^1^ Integrative Biology Oklahoma State University Stillwater Oklahoma USA

**Keywords:** nutrition, predation, spider, vertebrate prey

## Abstract

Herbivores and omnivores have been shown to regulate their intake of nutrients to a balance that maximizes fitness. Predators were traditionally believed to have less need for dietary regulation than herbivores, given the higher nutritional quality of animal tissue compared to plants. However, some predators, like spiders, may feed on diverse prey that could vary substantially in nutrient content and, hence, their potential quality as food items. This study compared the nutrient intake of Carolina wolf spiders ( *Hogna carolinensis* ) when they fed on cricket frogs (*Acris blanchardi)* and crickets ( *Gryllodes sigillatus* ). In diet trials, spiders were fasted prior to being offered a frog or cricket for consumption. Then, prey remains and nonconsumed (control) frog and cricket samples were analyzed for lipid, lean tissue, and elemental content. Results show that frogs and crickets vary substantially in the nutrients that they provide to spiders. Frogs offer less lipids but more lean tissue compared to crickets. Additionally, spiders consumed a greater mass of micronutrients when feeding on frogs compared to crickets. While some evidence suggests that lipids may be limited for some spider species, frogs may still be beneficial to spiders' diets because they offer an abundance of lean tissue. Future research should examine how environmental and physiological factors influence the nutritional quality of prey for predators.

## Graphical abstract

This study compared the nutrient intake of Carolina wolf spiders when they were fed on cricket frogs and crickets. Results show that frogs and crickets vary substantially in the nutrients that they provide to spiders. Furthermore, both prey are beneficial to the diet of the spiders.

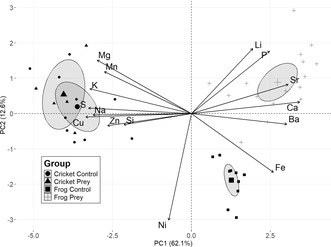

## 1 Introduction

Animals acquire nutrients using strategies adapted to their individual traits and ecological circumstances. For instance, some herbivores and omnivores have been shown to selectively choose their food source and thereby balance their nutrient intake depending on their nutritional needs (Mayntz et al. 2005; Jensen et al. 2011; Simpson and Raubenheimer 2012). Conversely, it is commonly thought that predators do not need to be as selective in their food choice because their prey are relatively higher quality; however, this is not always the case (Galef 1996; Jensen et al. 2011). For instance, spiders may consume prey items that are not always nutritionally balanced (Toft 2013). Moreover, prey items may vary widely in their nutritional content, and spiders can grow or reproduce better on some diets relative to others, highlighting the adaptability of spiders as versatile predators (Toft and Wise 1999; Mayntz et al. 2005; Toft 2013; Pekár et al. 2016; Wilder 2011; Herzog et al. 2023; Reeves et al. 2023).

Spiders are opportunistic generalist predators found in all terrestrial environments (Turnbull 1973) and are one of the largest groups of obligate carnivores (Li et al. 2021). Diets of spiders consist primarily of invertebrate prey, although species from dozens of families (e.g., Pisauridae, Theraphosidae, Dictynidae, Lycosidae) have been shown to consume vertebrate prey (Menin et al. 2005; Toledo 2005; Murray et al. 2016; Nyffeler et al. 2017; Baba et al. 2019; Badjedjea Babangenge et al. 2019; Nyffeler and Altig 2020; Folt and Guyer 2021; Nyffeler and Gibbons 2022; Wilder and Simpson 2022; Michalko and Pekár 2024). Indeed, many spider species can capture and secure vertebrate prey; in some cases, prey that is much larger than the spider itself (Menin et al. 2005; Nyffeler et al. 2017; Baba et al. 2019; Badjedjea Babangenge et al. 2019; Nyffeler and Gibbons 2022; Wilder and Simpson 2022; Michalko and Pekár 2024). For example, wolf spiders (Lycosidae) have morphological adaptations (e.g., leg structure, body size, & venom) that allow them to capture relatively large prey (Rovner 1980). In addition, some species of wolf spiders are larger in size and occupy moist habitats, which helps facilitate the capture and processing of vertebrate prey, such as amphibians (Nyffeler and Gibbons 2022; Wilder and Simpson 2022). Lycosidae have previously been observed eating vertebrate prey (Menin et al. 2005; Toledo 2005; Nyffeler et al. 2017; Nyffeler and Altig 2020; Nyffeler and Gibbons 2022) with multiple species of Lycosidae, including *Hogna carolinensis* , observed consuming frogs (Nyffeler and Altig 2020). Thus, including vertebrates in their diet could prove nutritionally beneficial for some spiders (Wise 1995, 2006), as these predators are generally prey‐limited in nature, and larger vertebrate prey may offer an alternative source of significant nutrients (Toft 2013; Nyffeler and Altig 2020; Nyffeler and Gibbons 2022; Wilder and Simpson 2022). However, studies on the nutritional aspects of spiders consuming vertebrate prey are limited.

The ability of spiders to extract nutrients from vertebrate prey can differ from invertebrates, likely due to the difference in structural composition between prey types (Wilder and Simpson 2022). For example, black widow spiders *(Latrodectus hasselti)* were less efficient at processing skinks than either locusts or beetles, suggesting that skinks are relatively low‐quality prey items (Wilder and Simpson 2022). Nonetheless, the opportunity to include skinks in their diet in addition to other invertebrate prey may offset the relatively lower quality of skinks (Wilder and Simpson 2022). Further, some invertebrate carnivores can selectively, by some unknown mechanism, acquire lipids and proteins from feeding on their prey to maintain a nutritionally balanced diet that is optimal for growth and reproduction, though this topic needs further investigation (Cuff et al. 2024). Hence, there may be potential for predators to manage nutrient intake from large vertebrate prey to match their current nutrient needs (Mayntz et al. 2005; Wilder 2011; Toft 2013).

Spiders feed through extra‐oral digestion, which allows them to break down nutrients in prey before ingestion (Overgaard and Wang 2012; Walter et al. 2017). This process involves regurgitating digestive enzymes that liquefy the prey's tissues and separates indigestible parts such as the exoskeleton (Overgaard and Wang 2012; Walter et al. 2017; Barnes et al. 2019). Extra‐oral digestion begins with the midgut releasing digestive enzymes from secretory cells that break down food (Overgaard and Wang 2012). Next, the mouth filters the intake of food particles that then travel down the pharynx to the muscular sucking stomach, which generates the pumping action needed for feeding and regurgitation (Overgaard and Wang 2012). Thus, extra‐oral digestion potentially enables spiders to selectively intake nutrients from prey (Mayntz et al. 2005; Overgaard and Wang 2012; Walter et al. 2017). In addition to extra‐oral digestion, some spiders use their chelicerae to masticate prey (Figure S1), such as wolf spiders, which may enable them to better extract nutrients from vertebrate prey (Wilder and Simpson 2022). Thus, differences in nutrient extraction between prey types are less likely due to simple structural differences. However, it remains unclear if there are differences in nutrient acquisition when spiders feed on invertebrate versus vertebrate prey.

This study was designed to analyze and compare the macro‐ and micronutrient content in the bodies of a common invertebrate and vertebrate prey and subsequent nutrient acquisition by Carolina wolf spiders ( *Hogna carolinensis* ) when feeding on these prey types. We examined the lipid, lean mass, and elements (Ba, Ca, Cu, Fe, Li, Mg, Mn, Ni, P, K, Na, Sr, S, Si, Zn) extracted by spiders from cricket frogs ( *Acris blanchardi* ) and banded crickets ( *Gryllodes sigillatus* ), both natural prey options for Carolina wolf spiders, to assess for potential differences in nutrients between invertebrate and vertebrate prey. The motivation for this study came from observing spiders eating tadpoles and the numerous observations of spider–frog interactions in the literature (Menin et al. 2005; Toledo 2005; Murray et al. 2016; Nyffeler et al. 2017; Baba et al. 2019; Badjedjea Babangenge et al. 2019; Nyffeler and Altig 2020; Folt and Guyer 2021; Nyffeler and Gibbons 2022), which led us to question whether frogs are viable prey for spiders. All developmental stages of a frog (egg, tadpole, metamorph, etc.) are susceptible to spider predation (Murray et al. 2016; Baba et al. 2019; Badjedjea Babangenge et al. 2019; Nyffeler and Altig 2020). However, postmetamorphic frogs were used in this study due to experimental design logistics and accessible laboratory housing conditions, and previous works highlight the commonality of this trophic relationship in natural settings (Menin et al. 2005; Toledo 2005; Murray et al. 2016; Nyffeler et al. 2017; Baba et al. 2019; Badjedjea Babangenge et al. 2019; Nyffeler and Altig 2020; Folt and Guyer 2021; Nyffeler and Gibbons 2022). Moreover, limited research has been conducted on how traits of vertebrate prey affect the nutritional quality for invertebrate predators (Wilder and Simpson 2022), making this study significant on a broader ecological scale as it compares nutrients between prey with different taxonomic identities and sizes. Furthermore, this study will expand current knowledge on nutrient regulation in an ecosystem through predatory–prey relationships.

## 2 Methods

Forty adult female Carolina wolf spiders were collected between 2100 h and 2400 h in Stillwater, Oklahoma, in May and June 2023. Only adult females were used since previous research has shown that reproduction may cause females to be selective of macronutrients from prey, whereas males do not consume as much food (Feng et al. 2022). Spiders were housed in 1.8 L (60 oz) plastic containers that contained a cardboard shelter and a moistened cotton ball. All spiders were fed one large cricket or mealworm every 5–8 days. Housing conditions were maintained at 25°C on a 15:9 light:dark cycle.

### 2.1 Diet Trials

Diet trials were conducted using banded crickets and Blanchard's cricket frogs. Frogs were collected from several ponds in Stillwater, Oklahoma while crickets were purchased from Josh's Frogs. Forty spiders, each tested once, were randomly placed individually into 9.5 L glass aquaria. The bottom of each aquarium was covered with filter paper and provided with a cardboard shelter, moistened cotton ball, and a petri dish of water to keep frogs moist. Spiders were fasted for 10–12 days (Wilder and Simpson 2022; Herzog et al. 2023) before the introduction of prey because frogs were only collected on certain days. Trials began at approximately 9 pm to optimize spider predation behavior, as this species is nocturnal. The forty spiders were placed into two groups of 20 to randomly be paired with either a cricket or a frog. Wet weight (±0.1 mg) and length (±1 mm) were recorded for all spiders, crickets, and frogs prior to the start of trials.

Trials lasted up to 72 h depending on when spiders finished consuming prey. Prey remains were then collected, weighed, and stored in 50 mL glass vials at room temperature before lipid extraction (see below). Prey remains were only collected once spiders stopped feeding on them, which was indicated when spiders discarded a small ball of their masticated prey (Figure S2). Prey remains consist mostly of exoskeletons, which are indigestible to spiders (Barnes et al. 2019). An additional 10 frogs and 10 crickets were collected as controls, their morphometric data collected as previously described, and then euthanized using MS‐222 (frogs) and by freezer (crickets) as done in previous studies (Windle et al. 2021; Kelly and Adam‐Granger 2020). All control frogs and crickets were stored at −18°C to be analyzed later.

### 2.2 Nutrient Analysis

Frog remains, cricket remains, and controls (i.e., unconsumed individuals of each species) were analyzed for total lipid and lean mass. Lipid content was determined gravimetrically following procedures modified from Cuff et al. (2021). Briefly, samples were dried to a constant weight at 60°C for 48 h, weighed, then ground in a Retsch Laboratory mixer mill MM400 at 30 Hz for 1–3 min until the sample was completely powdered. Five to eight milligrams of each sample were retained for elemental analysis (see below). The remaining mass from each sample was weighed and soaked in chloroform (5:1 chloroform: sample volume) for 24 h, then suctioned off and replaced with fresh chloroform for an additional 24 h. Chloroform was again removed, and samples were allowed to air dry for an additional 6 h. The weight of the sample (±0.1 mg) was recorded as the lean mass, and the difference in mass between pre‐ and postextraction was recorded as lipid.

### 2.3 Elemental Analysis

Five to eight milligrams from selected samples were analyzed to examine elemental content. Samples were analyzed for barium (Ba), calcium (Ca), copper (Cu), iron (Fe), lithium (Li), magnesium (Mg), manganese (Mn), nickel (Ni), phosphorus (P), potassium (K), sodium (Na), strontium (Sr), sulfur (S), silica (Si) and zinc (Zn) using inductively coupled plasma‐optical emission spectroscopy (ICP‐OES) following the methods of Prater et al. (2020). Estimates of the total mass of each element present in samples were calculated as the elemental concentrations from ICP (μg/g) times the dry mass of the prey sample.

### 2.4 Statistical Analysis

To calculate the amount of lipid and lean mass that spiders ingested when they fed on each prey, we estimated the amount of nutrients present in a prey item that was fed to a spider, measured the amount of nutrients in the prey remains, and calculated the difference. This process was the same as that used in past studies that have quantified spider nutrient intake (Wilder 2011). Lipid results for control frogs and crickets were used to generate predictive models of lipid from fresh body weights. These models, in turn, were used to estimate lipid mass in frogs and cricket prey that were fed to spiders before the spiders started feeding based on the live weight of these individuals before they were fed to the spiders.

To analyze the multivariate balance of elements consumed by spiders, we compared the set of observed elemental masses between control and prey remain groups using PERMANOVA and Principal Component Analysis (PCA). Prior to running PERMANOVA analyses, we performed an analysis of homogeneity of multivariate dispersions using *betadisper—permutest* (R package *vegan*) on a matrix of untransformed mass of each element (μg/g) to test for dispersion differences in overall elemental balance between groups (Oksanen et al. 2022). Given its robustness to overdispersion, we subsequently performed a PERMANOVA to assess location differences in the multivariate centroid between groups (i.e., differences in overall elemental balance).

For the PCA, we performed a centered log‐ratio (CLR) transformation on the matrix containing the masses of all 15 elements and performed a PCA on the transformed matrix using Bray‐Curtis distances (Greenacre 2021). We subsequently extracted values of the principal components explaining the highest amount of variation in all dimensions (PC1 and PC2) and tested the effects of prey species (frog or cricket) and consumption (consumed or not consumed) using a 3‐way ANOVA.

Finally, we tested for differences in the mass of individual elements between prey species and consumption treatment. We used Levene's test to examine potential differences in variance of grouping by prey species (frog or cricket) and consumption (consumed or not consumed). Subsequently, we used ANOVA to test differences between group means with homogenous variance, and Welch's ANOVA to test differences between groups with heterogeneous variance.

## 3 Results

### 3.1 Predictive Macronutrient Models

Average weight and length, respectively, were 1973.5 ± 44.5 mg and 26.05 ± 0.2 mm for spiders, 292.6 ± 13.6 mg and 16.9 ± 0.3 mm for crickets, and 1100.8 ± 34.2 mg and 23.3 ± 0.5 mm for frogs. The fresh weight of control frogs and crickets did not differ from their counterparts that were fed to spiders (Table 1). Lipid weights of control frogs were used to produce a model for predicting lipid in frogs used as prey in diet trials based on the fresh weight of the animal (Equation 1). For lipid, a nonlinear model was chosen over a linear model (*R* ^2^ = 0.02) because the nonlinear model better aligned with the data. The lipid model produced was as follows:

(1) $$y=-1.20{\text{4E}}^{-4}x^{2}+0.272x-138.298\;\;\;\left(R^{2}=0.52\right)$$

where *y* = the predicted lipid mass (mg) and *x* = fresh body weight (mg). Likewise, data from control crickets were used to produce the lipid model in Equation (2) where *y* = lipid mass (mg) and *x* = fresh body weight (mg).

(2) $$y=0.0231x+8.250\;\;\;\left(R^{2}=0.14\right)$$

**TABLE 1:** Mean (±SE) weight (mg) of frog and cricket samples consumed (prey) and not consumed (control) by Carolina wolf spiders.

Sample type	Frogs		Crickets	
	Control	Prey	Control	Prey
Fresh	1022.3 ± 59.0	1153.0 ± 36.8	284.3 ± 30.9	297.2 ± 13.0
Dry	244.6 ± 16.5	132.4 ± 16.9	87.8 ± 14.9	38.3 ± 5.5
Lipid	10.6 ± 1.1	14.0 ± 1.2	14.8 ± 1.9	3.6 ± 0.8
Lean	233.9 ± 16.7	118.5 ± 16.5	73.0 ± 14.3	34.7 ± 4.7

*Note:* Weights include before predation trials (fresh), after samples were baked (dry), lipid in the dried sample (lipid), and weight after lipid extraction (lean). Sample size (*n*) = 10 each for control frogs and crickets, 15 for prey frogs, and 18 for prey crickets.

Lean weights of control animals were used to produce models to predict lean tissue in frogs (Equation 3) and crickets (Equation 4) based on their wet mass, where *y* = lean mass (mg) and *x* = fresh body weight (mg).

(3) $$y=0.2794x-51.747\;\;\;\left(R^{2}=0.97\right)$$

(4) $$y=0.4499x-54.953\;\;\;\left(R^{2}=0.94\right)$$

### 3.2 Macronutrient Consumption Analysis

Spiders consumed more lipid from crickets than frogs (Table 2). Spiders consumed essentially none of the 14 mg of lipid available per frog (Tables 1 and 2). Conversely, spiders consumed over 11 mg of lipid from crickets, which was over 75% of the available lipid in cricket samples (Table 2). Due to the variability in the lipid predictive model, negative values in Table 2 were inferred to represent little to no acquisition of lipids.

**TABLE 2:** Mean (±SE) estimates for mass (mg) of lipid and lean tissue predicted to be in samples before consumption by Carolina wolf spiders.

	Frog	Cricket	*t*	*p*
Predicted mass				
Lipid	13.5 ± 0.8	15.1 ± 0.3	2.10	0.07
Lean tissue	270.4 ± 10.3	78.7 ± 5.9	2.07	< 0.0001
Mass consumed				
Lipid	−0.5 ± 1.3	11.5 ± 0.7	2.07	< 0.0001
Lean tissue	151.9 ± 16.2	44.1 ± 5.6	2.11	< 0.0001
% Consumption				
Lipid	−8.1 ± 9.8	76.7 ± 5.0	2.08	< 0.0001
Lean tissue	56.4 ± 5.9	55.8 ± 5.0	2.05	0.94

*Note:* Mean (±SE) estimates for mass (mg) and percentage of lipid and lean tissue consumed by spiders. Sample size (*n*) was 15 for frogs and 18 for crickets.

Average lean tissue consumed from frogs was about three‐fold greater than that from crickets, which paralleled initial predicted differences between frogs and crickets (Table 2). However, spiders consumed the same percentage of available lean mass from frogs compared to crickets (Table 2).

### 3.3 Elemental Analysis

The results of the analysis of homogeneity of multivariate dispersions using *betadisper* and *permutest* (Oksanen et al. 2022) performed on untransformed elemental masses (i.e., μg) indicated that multivariate dispersions did not differ between species (df = 1; residual df = 39; *F* = 2.3; permutations = 999; *p* = 0.1) or prey treatment (df = 1; residual df = 39; *F* = 2.0; permutations = 999; *p* = 0.2). The PERMANOVA computed using these data detected significant dissimilarity between species (df = 1; residual df = 37; *F* = 205; *R* ^2^ = 0.8; *p* = 0.001) and prey treatment (df = 1; residual df = 37; *F* = 13.4; *R* ^2^ = 0.05; *p* = 0.001) but not the two‐way interaction. Thus, these results rejected the null hypothesis that the locations of species and treatment centroids do not differ in multivariate space.

**FIGURE 1:**
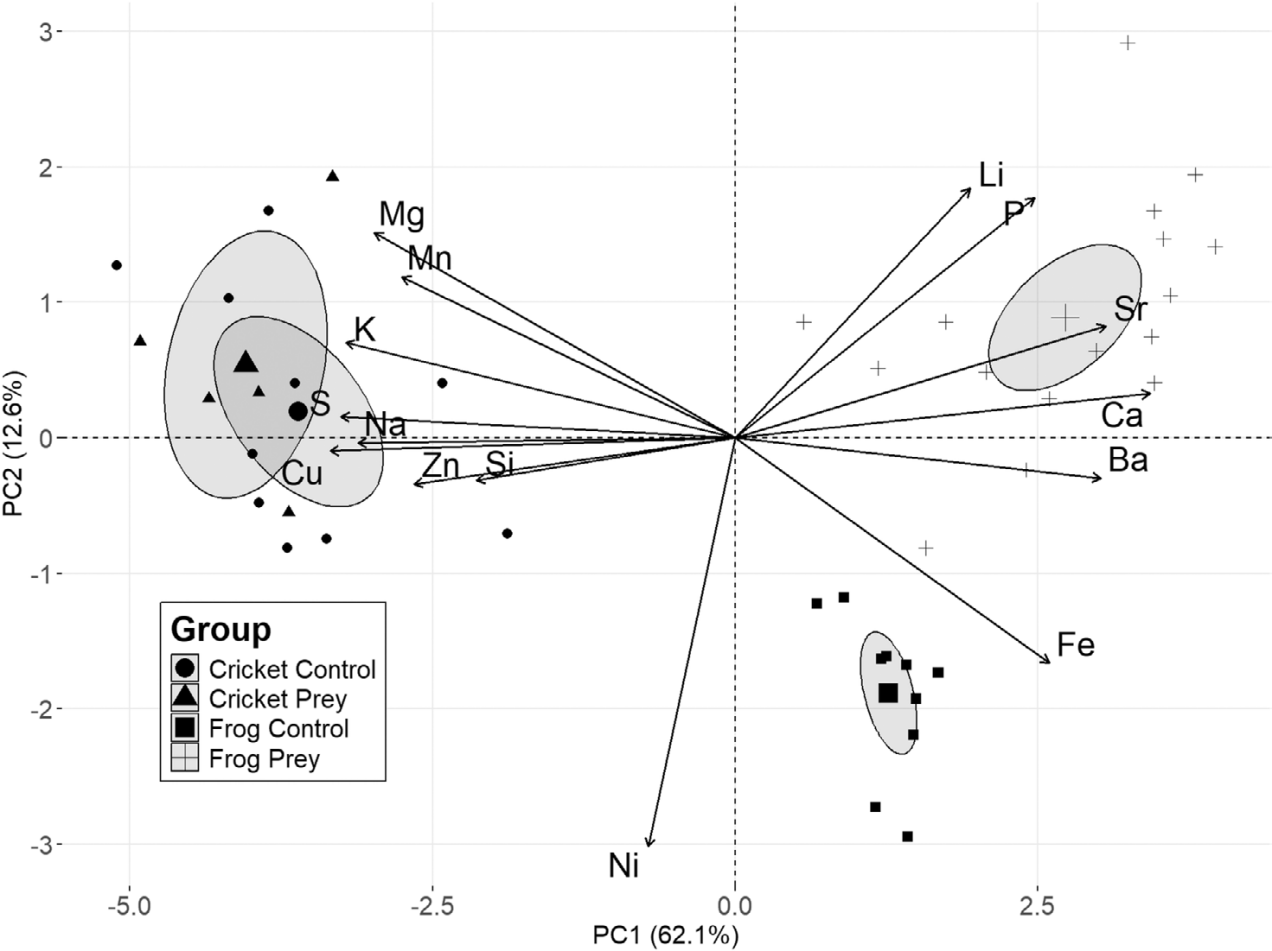
Principal component analysis performed on centered log‐ratio‐transformed masses of 15 elements in control and prey crickets and frogs.

The first two components extracted from the Principal Component Analysis performed on centered log‐ratio‐transformed elemental masses explained 62.1% (PC1) and 12.6% (PC2) of the original variance of the dataset (Figure 1). On the first component (PC1), the elements Ba, Ca, Fe, P, and Sr displayed positive loading scores > |0.2|, and the elements Cu, K, Mg, Mn, Na, S, and Zn displayed negative loading scores > |0.2| (Table 3). On the second component (PC2), the elements Li, Mg, Mn, and P displayed positive loading scores > |0.2|, and Fe and Ni displayed negative loading scores > |0.2| (Table 3).

**TABLE 3:** Loadings for individual elements on principal components 1 and 2.

Element	PC1	PC2
Ba	**0.28**	−0.061
Ca	**0.32**	0.067
Cu	**−0.31**	−0.020
Fe	**0.24**	**−0.34**
K	**−0.30**	0.14
Li	0.18	**0.38**
Mg	**−0.28**	**0.31**
Mn	**−0.25**	**0.24**
Na	**−0.29**	−0.0077
Ni	−0.066	**−0.62**
P	**0.23**	**0.36**
S	**−0.30**	0.032
Si	−0.197	−0.064
Sr	**0.28**	0.17
Zn	**−0.24**	−0.070

*Note:* Elements with loading > |0.2| for each axis are bolded.

ANOVAs computed on extracted PC axis values indicated that species identity (df = 1; residual df = 37; *F* = 413; *p* < 0.0001), treatment (df = 1; residual df = 37; *F* = 77.2; *p* < 0.0001), and the interaction between species * treatment (df = 1; residual df = 37; *F* = 11.2; *p* = 0.0002) affected mean PC1 scores. Additionally, species identity affected mean PC2 scores (df = 1; residual df = 37; *F* = 13.6; *p* = 0.0007), as well as treatment (df = 1; residual df = 37; *F* = 40.8; *p* < 0.0001) and the interaction between species * treatment (df = 1; residual df = 37; *F* = 18.6; *p* = 0.0001). These statistical results appear to describe the patterns in the PCA plot in which there is greater separation between control versus prey frogs than between control versus prey crickets, which indicates that spiders extracted more micronutrient elements from frogs than crickets (Figure 1).

Levene's test indicated that the variance of some individual elements differed between control and prey samples of both species (Tables S1 and S2). The elements Fe and P displayed differences in variance between control and prey crickets, but Ba, Li, Na, Ni, and Zn displayed differences in variance between control and prey frogs (Table S1). Elements in control samples also differed in variance between species, including Ba, Ca, Fe, Ni, P, Si, Sr, and Zn, where differences in elemental variance between species of prey samples occurred in Ba, Fe, Mg, Ni, P, S, and Sr (Table S2).

We also compared individual elements within and between species. Control frogs displayed higher mean masses of all elements compared to control crickets, in line with their larger body size (Tables 4 and S3). Prey frogs contained higher mean masses of most elements compared to prey crickets but contained similar amounts of Cu, Mn, and Zn (Tables 4 and S3). Control frogs contained higher mean masses of Ba, Cu, Fe, K, Mg, Mn, Na, Ni, S, Si, and Zn than the remains of frog prey, indicating that significant quantities of these elements were ingested by spiders feeding on the frogs (Tables 4 and S4). Control crickets had higher mean masses of Fe, P, and S than cricket prey (Tables 4 and S4).

**TABLE 4:** Mean (±SE) estimates of the elemental masses (μg) of frog and cricket samples consumed (prey remains) and not consumed (whole prey).

Element	Cricket		Frog	
	Whole prey	Prey remains	Whole prey	Prey remains
Barium (Ba)	1.6 ± 0.4	1.7 ± 0.4	36.9 ± 3.1	23.6 ± 1.4
Calcium (Ca)	288.1 ± 68.7	230.3 ± 57.0	10306.5 ± 550.8	10463.0 ± 747.6
Copper (Cu)	7.1 ± 1.2	7.3 ± 1.8	16.4 ± 2.0	6.3 ± 1.1
Iron (Fe)	2.5 ± 0.5	0.3 ± 0.2	137.4 ± 25.6	51.9 ± 10.7
Potassium (K)	974.6 ± 150.4	753.4 ± 115.2	2628.9 ± 143.3	1530.4 ± 179.8
Lithium (Li)	0.4 ± 0.2	0.4 ± 0.3	1.1 ± 0.1	1.9 ± 0.3
Magnesium (Mg)	149.6 ± 37.8	106.6 ± 11.0	510.1 ± 55.3	345.5 ± 37.8
Manganese (Mn)	2.8 ± 0.9	3.4 ± 0.7	6.0 ± 0.9	4.2 ± 0.4
Sodium (Na)	510.1 ± 115.0	304.2 ± 74.2	1664.9 ± 72.3	837.6 ± 132.0
Nickel (Ni)	0.1 ± 0.04	0.1 ± 0.02	2.8 ± 0.4	0.3 ± 0.04
Phosphorus (P)	755.4 ± 133.4	358.0 ± 25.8	6386.8 ± 357.1	5741.5 ± 435.2
Sulfur (S)	535.1 ± 83.1	276.6 ± 32.5	1709.3 ± 110.8	878.8 ± 141.5
Silicon (Si)	6.5 ± 1.4	3.9 ± 0.7	35.0 ± 4.0	18.2 ± 3.0
Strontium (Sr)	1.3 ± 0.3	1.0 ± 0.3	20.9 ± 1.9	25.3 ± 2.8
Zinc (Zn)	41.0 ± 8.4	42.9 ± 6.8	195.1 ± 53.8	70.2 ± 12.1

*Note:* Elemental masses were calculated from ICP‐OES analysis. Sample size (*n*) was 10 each for control frogs and crickets, 16 for consumed frogs, and 5 for consumed crickets.

## 4 Discussion

Our results suggest that Carolina wolf spiders may acquire different balances of nutrients from vertebrate and invertebrate prey. Spiders consumed similar proportions of available lean tissue from both prey items but were able to consume more mass of lean tissue from frogs due to their larger body size. Conversely, frogs and crickets had similar masses of available lipids (Frog:10.6 mg, Cricket:14.8 mg) regardless of the overall body size differences between them (Table 1). However, spiders extracted essentially none of the lipids present in frogs and most of the lipids present in crickets (Table 2). Spiders also ingested a variety of micronutrients from frogs. The diet requirements of spiders are not well known for most species. Yet, given that frogs should represent additional prey opportunities, the consumption of these vertebrates and the large amounts of lean tissue and micronutrients that they provide should benefit wolf spiders. Micronutrients are influential to the physiological processes of an animal (Simpson and Raubenheimer 2012); thus, a shortfall of micronutrients can have detrimental effects on the development and fitness of organisms that can lead to greater cascading effects that disrupt the ecosystem (Kaspari 2021). Furthermore, wolf spiders can preferentially assimilate and excrete certain micronutrients, though the extent to how and which micronutrients affect spider fitness still needs to be explored (Herzog et al. 2023).

Spiders are believed to be able to selectively intake nutrients through extra‐oral digestion (Mayntz et al. 2005; Overgaard and Wang 2012; Walter et al. 2017); however, previous research suggests that differences in body structures between invertebrates and vertebrates may influence the efficiency of spider nutrient extraction (Wilder and Simpson 2022). For instance, vertebrates exhibit greater compartmentalization of their bodies due to their closed circulatory system, while the open circulatory system of invertebrates may allow for easier movement of digestive enzymes within the prey's body (Evans 2022; Wilder and Simpson 2022). These differences in body structure may be especially important for spiders that feed on prey through small openings on the prey body, such as black widows (Wilder and Simpson 2022). However, Carolina wolf spiders use mastication to feed, grinding their prey into small pieces. This process likely minimizes the impact of prey body structure on nutrient extraction. Indeed, Carolina wolf spiders extracted a significant amount of lean tissue from frogs (Tables 1 and 2).

Crickets and frogs differed both in their body nutrient composition and in the availability of those nutrients to the spiders that fed on them. Interestingly, while frogs and crickets had a similar mass of lipids in their bodies, spiders did not appear to consume any of the lipids present in frogs. This may be due to the disproportionate lipid‐to‐body size ratio between prey, making the lipid content relatively insignificant compared to the total consumable tissue in frogs. Lipids are important to spiders because they may use lipids as energy reserves to endure long periods of starvation (Overgaard and Wang 2012); however, it is unclear if spiders select prey according to the lipid reserves of the prey. It would be interesting to study this further to determine if spiders were unable or unwilling to consume lipids present in the frog body.

Our results also showed that crickets and frogs possess different micronutrient compositions in their bodies (Figure 1). Frogs showed significantly higher masses of all elements than crickets. Some of these differences may be explained by body size, and others may be explained by differences in the anatomy of vertebrates and invertebrates. For example, higher masses of Ca and P in frogs may be due to bone, while higher masses of Fe may be due to hemoglobin in the red blood cells of vertebrates (Howard 1951; Hagner‐Holler et al. 2004; Chen and Paw 2012; Wilder and Simpson 2022).

There may also be differences in the digestibility of different elements in vertebrates versus invertebrates. Some pools of elements may be less digestible, including elements in the bones of vertebrates and the exoskeleton of invertebrates. For example, despite frogs having a large quantity of Ca, spiders did not appear to consume any of it. The bones in frogs likely contributed to the high Ca levels, but we observed that these bones were usually left behind in carcass remains after spiders finished consuming vertebrate prey (Nyffeler and Gibbons 2022). Our data indicate that Ca mass in both frog whole bodies and prey remains is similar. This suggests that spiders do not acquire a substantial amount of Ca from ingesting frogs. Conversely, Wilder and Simpson (2022) discovered that redback spiders consumed Ca from vertebrate prey, specifically skinks, indicating that some spiders can extract Ca from the soft tissues of some vertebrates. Future research should investigate mechanisms of Ca acquisition and its potential importance in spiders.

Spiders acquired a significant amount of mass from additional elements (Ba, Ca, Fe, Ni, Si, Sr, Zn) in frogs compared to crickets (Tables 4 and S3). However, this disparity may be due to the lower sample size of crickets in the elemental analysis or the physiological differences between frogs and crickets. The exoskeleton of crickets can complicate nutrient acquisition for spiders. Previous research suggests that the elemental composition of whole prey may not accurately represent nutrients available to predators due to the indigestibility of the exoskeleton (Herzog et al. 2023; Reeves et al. 2023). Hence, it is important to consider the digestibility of nutrients when analyzing the whole‐body contents of prey.

While there are several records of frog predation by spiders, studies have shown that frogs represent a small portion of spiders' diet as spiders primarily feed on insects and arthropod prey, although this may be species specific (Nyffeler and Altig 2020). Indeed, crickets can provide a good source of proteins, lipids, and vitamins (Magara et al. 2020). Spiders frequently face periods of starvation, which makes capturing any prey highly beneficial (Jensen et al. 2011). Hence, vertebrates may be beneficial to spiders' diets because they offer a surplus of nutrients due to their larger size (Nyffeler and Gibbons 2022; Wilder and Simpson 2022). Our study shows that spiders consume prey with varying nutritional compositions. Therefore, by feeding on a variety of prey as opportunistic generalist predators, spiders can expose themselves to a diverse range of nutrients. Although the requirements of spiders for certain micronutrients remain poorly understood, previous work has shown that dietary mixing or diet diversity can benefit spider fitness (Wilder 2011). Hence, even if one prey is imbalanced in nutrient content (i.e., high in some nutrients and low in others) spiders can balance their diet and maximize fitness by feeding on different types of prey that vary in their nutrient concentrations.

Organismal nutrient composition can fluctuate depending on environmental conditions and physiological state (Back and King 2013; Oloo et al. 2020). Diet, age, and exposure to contaminants can influence the nutritional quality of crickets (Oloo et al. 2020; Labu et al. 2022). Likewise with frogs, nutrient composition may depend upon sex, as female frogs normally contain more lipids since fats facilitate successful reproduction (Bruscalupi et al. 1989). The sex of the frog was not recorded in this study, and the minimal amount of lipid present in frog prey could be explained primarily using male frogs. Additionally, the predictive model used for lipid mass in frogs may vary from a nonlinear model depending on the sex and reproductive state of the frog. Furthermore, the nutritional composition of frogs can also fluctuate with developmental stage (egg, tadpole, metamorph, etc.), which can all be preyed upon by spiders (Ferrie et al. 2014; Nyffeler and Altig 2020). Future experimental designs could examine how these internal and external factors influence the nutrient composition of prey for predators. This would provide insight into the nutritional significance of prey in a predator's diet and assess its potential impact on the predator's fitness. Indeed, the global decline of amphibians disrupts predator–prey relationships, significantly altering animal communities (Houlahan et al. 2000; Blaustein et al. 2010). Thus, understanding the nutritional value of amphibians to their predators is essential for assessing their roles in food webs. Therefore, further research is needed to explore the nutritional significance of amphibians to predators, as this would provide a clearer understanding of the ecological impacts on spiders resulting from amphibian population changes.

## Author Contributions

**Chloe G. Hunsucker:** conceptualization (equal), data curation (equal), formal analysis (equal), investigation (equal), methodology (equal), validation (equal), visualization (equal), writing – original draft (equal), writing – review and editing (equal). **Colton Herzog:** conceptualization (equal), data curation (equal), formal analysis (equal), investigation (equal), methodology (equal), validation (equal), visualization (equal), writing – original draft (equal), writing – review and editing (equal). **Jamie T. Reeves:** conceptualization (equal), data curation (equal), formal analysis (equal), funding acquisition (equal), methodology (equal), software (equal), validation (equal), visualization (equal), writing – original draft (equal), writing – review and editing (equal). **Shawn M. Wilder:** conceptualization (equal), data curation (equal), formal analysis (equal), funding acquisition (equal), investigation (equal), methodology (equal), project administration (equal), resources (equal), supervision (equal), validation (equal), visualization (equal), writing – original draft (equal), writing – review and editing (equal). **Scott T. McMurry:** conceptualization (equal), data curation (equal), formal analysis (equal), funding acquisition (equal), investigation (equal), methodology (equal), project administration (equal), resources (equal), software (equal), supervision (equal), validation (equal), visualization (equal), writing – original draft (equal), writing – review and editing (equal).

## Conflicts of Interest

The authors declare no conflicts of interest.

## Supporting information


Figure S1.

Figure S2.

Data S1.

## Data Availability

The data that support the findings of this study are openly available in Figshare at (Wilder 2024) https://doi.org/10.6084/m9.figshare.c.7517703.v1.
