# Supplementary figures and images for: Comparing Nutrient Intake by Wolf Spiders (Hogna carolinensis) Consuming Frogs (Acris blanchardi) and Crickets (Gryllodes sigillatus)
Source: Ecol Evol. 2025 Mar 2;15(3):e71045. doi: 10.1002/ece3.71045 (PMC11872198; doi:10.1002/ece3.71045)

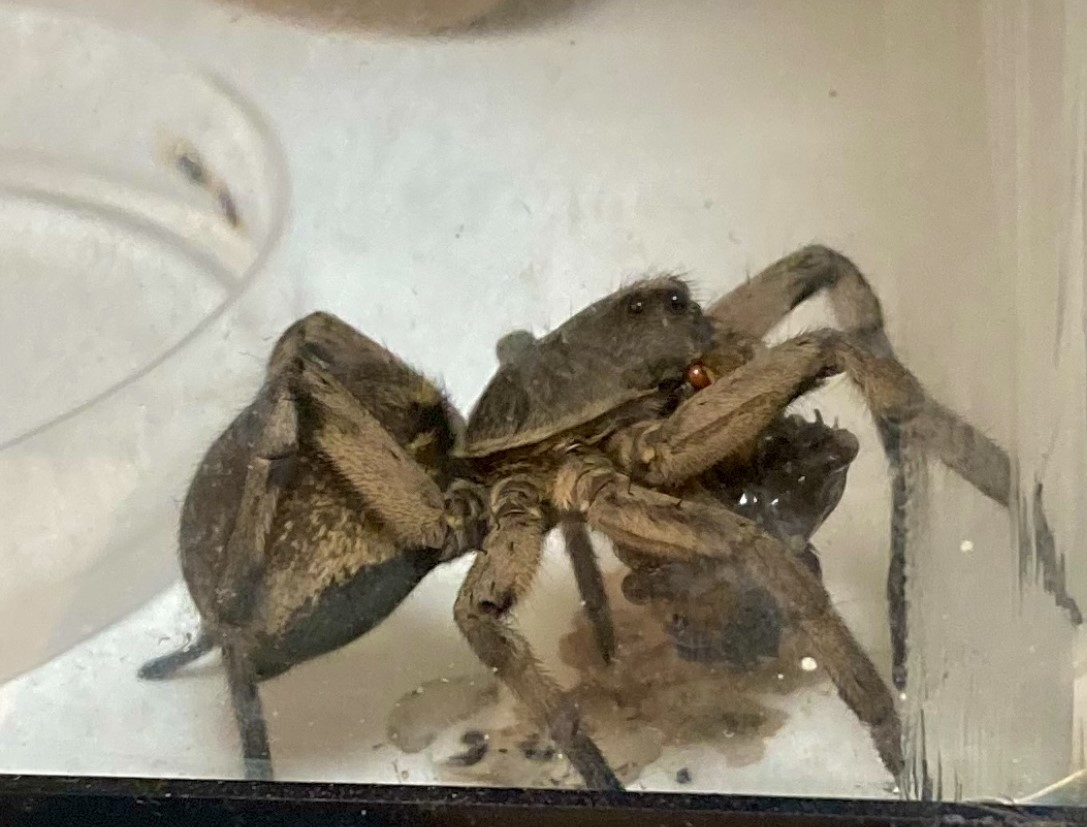

Supplement: Supplementary file 1 — Figure S1. [file ECE3-15-e71045-s001.tiff]

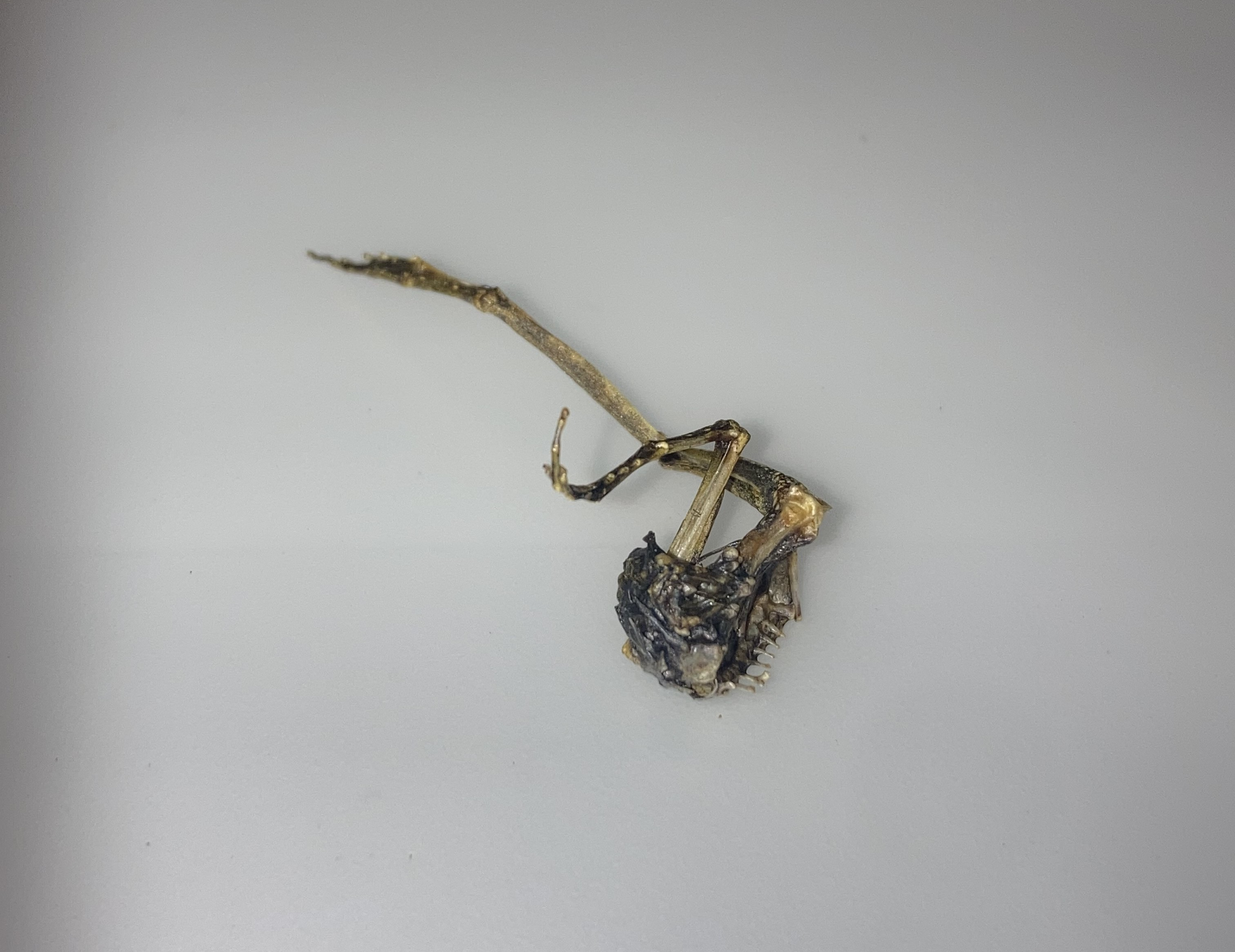

Supplement: Supplementary file 2 — Figure S2. [file ECE3-15-e71045-s003.tiff]
