# Supplementary material for: Comparing Nutrient Intake by Wolf Spiders (Hogna carolinensis) Consuming Frogs (Acris blanchardi) and Crickets (Gryllodes sigillatus)
Source: Ecol Evol. 2025 Mar 2;15(3):e71045. doi: 10.1002/ece3.71045 (PMC11872198; doi:10.1002/ece3.71045)
Supplement: Supplementary file 3 — Data S1. [file ECE3-15-e71045-s002.docx]

Supplementary Data


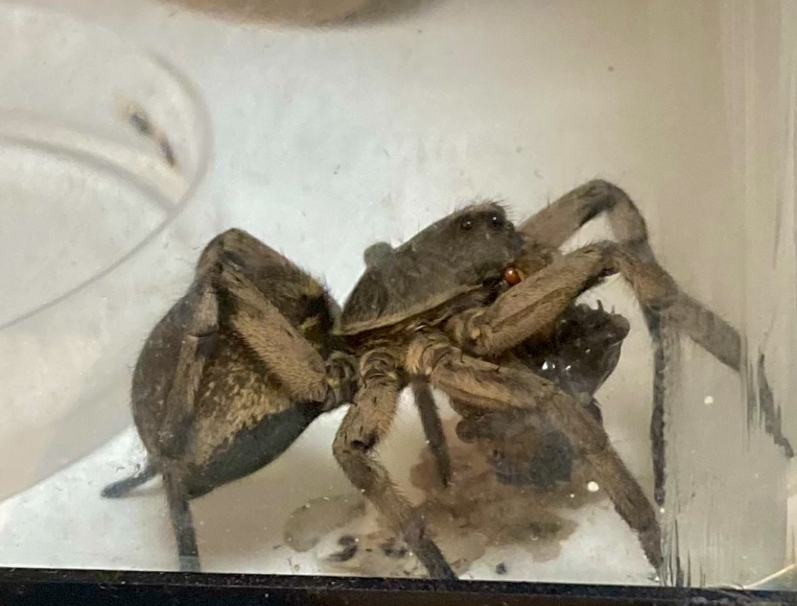


Figure S1. Carolina wolf spider (*Hogna carolinensis*) feeding on a cricket frog (*Acris blanchardi*).


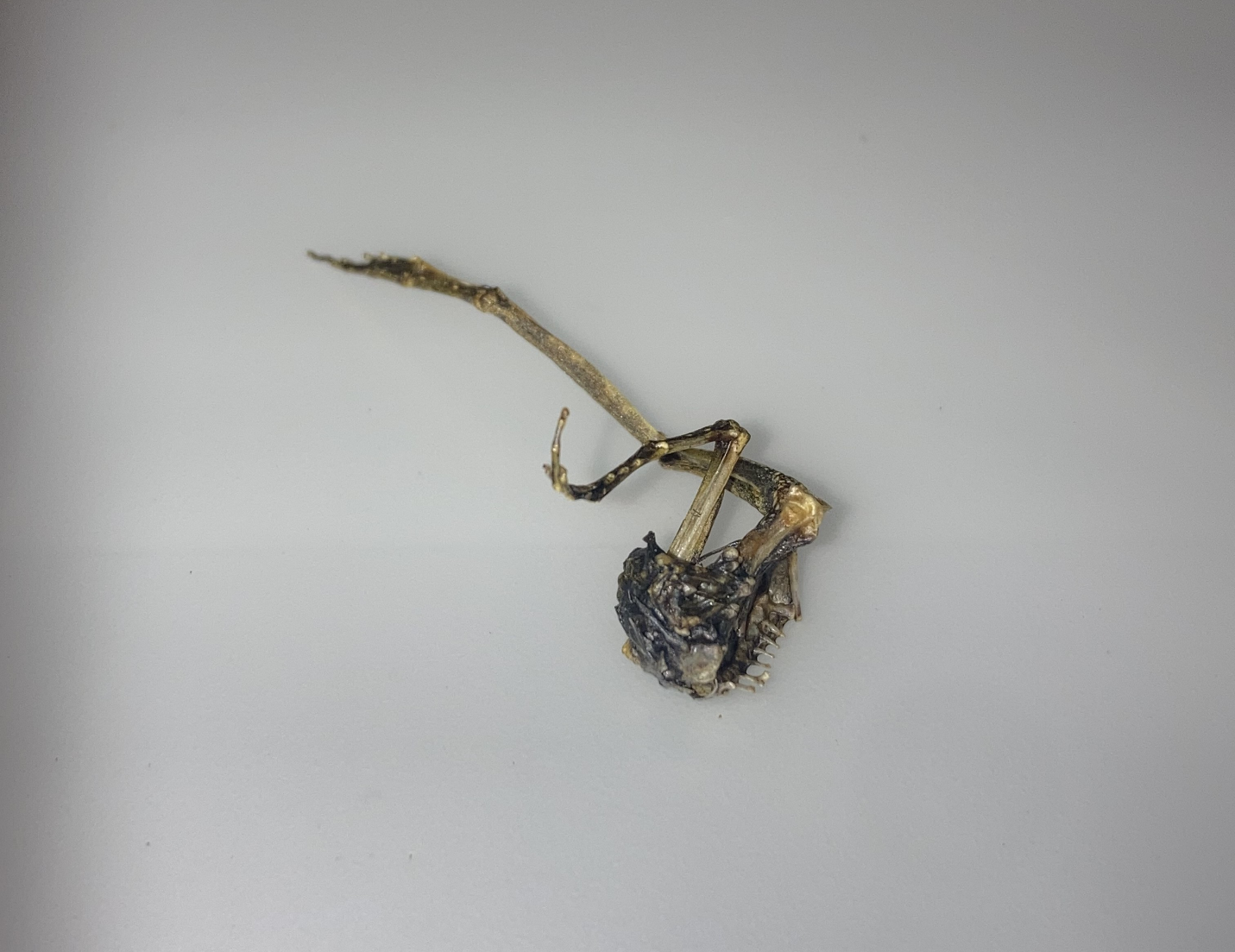
Figure S2. Prey remains of a cricket frog (*Acris blanchardi)* that was consumed by a Carolina wolf spider (*Hogna carolinensis*). This is what a typical prey remains looks like the day after feeding.

Table S1. Levene’s test statistics for heteroscedasticity in elemental content between prey treatments of each species. Elements with p < 0.05 are considered to have differing variance among groups.

|  | **Cricket** | | | |  | **Frog** | | | |
| --- | --- | --- | --- | --- | --- | --- | --- | --- | --- |
| **Element** | **df** | **rdf** | **F Statistic** | **p** |  | **df** | **rdf** | **F Statistic** | **p** |
| Ba | 1 | 13 | 1.29 | 0.3 |  | 1 | 24 | 4.63 | 0.04 |
| Ca | 1 | 13 | 0.59 | 0.5 |  | 1 | 24 | 0.81 | 0.4 |
| Cu | 1 | 13 | 0.37 | 0.6 |  | 1 | 24 | 2.44 | 0.1 |
| Fe | 1 | 13 | 10.5 | 0.006 |  | 1 | 24 | 3.76 | 0.06 |
| K | 1 | 13 | 0.96 | 0.3 |  | 1 | 24 | 2.19 | 0.2 |
| Li | 1 | 13 | 0.036 | 0.9 |  | 1 | 24 | 17.9 | 0.0003 |
| Mg | 1 | 13 | 2.14 | 0.2 |  | 1 | 24 | 0.013 | 0.9 |
| Mn | 1 | 13 | 0.43 | 0.5 |  | 1 | 24 | 0.36 | 0.6 |
| Na | 1 | 13 | 0.48 | 0.5 |  | 1 | 24 | 5.3 | 0.03 |
| Ni | 1 | 13 | 0.97 | 0.3 |  | 1 | 24 | 13.4 | 0.001 |
| P | 1 | 13 | 5.66 | 0.03 |  | 1 | 24 | 0.78 | 0.4 |
| S | 1 | 13 | 2.97 | 0.1 |  | 1 | 24 | 2.85 | 0.1 |
| Si | 1 | 13 | 2 | 0.2 |  | 1 | 24 | 0.33 | 0.6 |
| Sr | 1 | 13 | 0.38 | 0.6 |  | 1 | 24 | 2.17 | 0.2 |
| Zn | 1 | 13 | 1.29 | 0.3 |  | 1 | 24 | 12 | 0.002 |

Table S2. Levene’s test statistics for evaluation of heteroscedasticity in mass of individual elements between species. Elements with p < 0.05 are considered to have differing variance among groups.

|  | **Control** | | | |  | **Prey** | | | | |
| --- | --- | --- | --- | --- | --- | --- | --- | --- | --- | --- |
| **Element** | **df** | **rdf** | **F Statistic** | **p** |  | **df** | **rdf** | **F Statistic** | **p** | |
| Ba | 1 | 18 | 20.4 | 0.0003 |  | 1 | 19 | 8.25 | 0.01 | |
| Ca | 1 | 18 | 11.1 | 0.004 |  | 1 | 19 | 3.51 | | 0.08 |
| Cu | 1 | 18 | 3.17 | 0.09 |  | 1 | 19 | 0.034 | 0.9 | |
| Fe | 1 | 18 | 13.2 | 0.002 |  | 1 | 19 | 4.45 | 0.05 | |
| K | 1 | 18 | 0.0064 | 0.9 |  | 1 | 19 | 3.39 | 0.08 | |
| Li | 1 | 18 | 3.31 | 0.09 |  | 1 | 19 | 2.27 | 0.1 | |
| Mg | 1 | 18 | 0.84 | 0.4 |  | 1 | 19 | 8.49 | 0.01 | |
| Mn | 1 | 18 | 0.096 | 0.8 |  | 1 | 19 | 0.041 | 0.8 | |
| Na | 1 | 18 | 0.29 | 0.6 |  | 1 | 19 | 3.78 | 0.07 | |
| Ni | 1 | 18 | 9.35 | 0.007 |  | 1 | 19 | 4.45 | 0.05 | |
| P | 1 | 18 | 5.85 | 0.03 |  | 1 | 19 | 4.79 | 0.04 | |
| S | 1 | 18 | 0.79 | 0.4 |  | 1 | 19 | 7.95 | 0.01 | |
| Si | 1 | 18 | 8.8 | 0.008 |  | 1 | 19 | 3.3 | 0.08 | |
| Sr | 1 | 18 | 27.8 | < 0.0001 |  | 1 | 19 | 6.99 | 0.02 | |
| Zn | 1 | 18 | 11.3 | 0.003 |  | 1 | 19 | 2.96 | 0.1 | |

Table S3. Test statistics for ANOVA and Welch’s ANOVA evaluating differences in the mean mass of individual elements between species of each treatment type (control or prey). Elements with p < 0.05 are considered to have differing mean mass (µg) among groups. (*) under column W indicates use of Welch’s ANOVA for that element.

|  | **Control** | | | | |  | **Prey** | | | | |
| --- | --- | --- | --- | --- | --- | --- | --- | --- | --- | --- | --- |
| **Element** | **df** | **rdf** | **W** | **F-statistic** | **p** |  | **df** | **rdf** | **W** | **F-statistic** | **p** |
| Ba | 1 | 9 | * | 130 | < 0.0001 |  | 1 | 17 | * | 223 | < 0.0001 |
| Ca | 1 | 9 | * | 326 | < 0.0001 |  | 1 | 19 |  | 56.5 | < 0.0001 |
| Cu | 1 | 18 |  | 15.7 | 0.0009 |  | 1 | 19 |  | 0.24 | 0.6 |
| Fe | 1 | 9 | * | 27.7 | 0.0005 |  | 1 | 15 | * | 23.3 | 0.0002 |
| K | 1 | 18 |  | 63.4 | < 0.0001 |  | 1 | 19 |  | 5.45 | 0.03 |
| Li | 1 | 18 |  | 7.37 | 0.01 |  | 1 | 19 |  | 6.41 | 0.02 |
| Mg | 1 | 18 |  | 29.0 | < 0.0001 |  | 1 | 17 | * | 36.8 | < 0.0001 |
| Mn | 1 | 18 |  | 6.66 | 0.02 |  | 1 | 19 |  | 1.01 | 0.3 |
| Na | 1 | 18 |  | 72.3 | < 0.0001 |  | 1 | 19 |  | 4.80 | 0.04 |
| Ni | 1 | 9 | * | 56.2 | < 0.0001 |  | 1 | 19 | * | 17.5 | 0.0005 |
| P | 1 | 18 |  | 218 | < 0.0001 |  | 1 | 15 | * | 152 | < 0.0001 |
| S | 1 | 18 |  | 71.9 | < 0.0001 |  | 1 | 16 | * | 17.2 | 0.0007 |
| Si | 1 | 11 | * | 45.8 | < 0.0001 |  | 1 | 19 |  | 6.79 | 0.02 |
| Sr | 1 | 9 | * | 101 | < 0.0001 |  | 1 | 15 | * | 75.6 | < 0.0001 |
| Zn | 1 | 9 | * | 8.01 | 0.02 |  | 1 | 19 |  | 1.48 | 0.2 |

Table S4. Test statistics for ANOVA and Welch’s ANOVA evaluating differences in the mean concentration of individual elements among tested groups (control and prey frogs and crickets). Elements with p < 0.05 are considered to have differing mean mass (µg) among groups. (*) under column W indicates use of Welch’s ANOVA for that element.

|  | **Cricket** | | | | |  | **Frog** | | | | |
| --- | --- | --- | --- | --- | --- | --- | --- | --- | --- | --- | --- |
| **Element** | **df** | **rdf** | **W** | **F-statistic** | **p** |  | **df** | **rdf** | **W** | **F-statistic** | **p** |
| Ba | 1 | 13 |  | 0.039 | 0.8 |  | 1 | 13 | * | 15.4 | 0.002 |
| Ca | 1 | 13 |  | 0.30 | 0.6 |  | 1 | 24 |  | 0.022 | 0.9 |
| Cu | 1 | 11 | * | 0.019 | 0.9 |  | 1 | 24 |  | 23.6 | < 0.0001 |
| Fe | 1 | 13 |  | 13.8 | 0.004 |  | 1 | 24 |  | 12.5 | 0.002 |
| K | 1 | 13 |  | 0.92 | 0.4 |  | 1 | 24 |  | 18.6 | 0.0002 |
| Li | 1 | 13 |  | 0.0011 | 1.0 |  | 1 | 16 | * | 6.66 | 0.02 |
| Mg | 1 | 13 |  | 0.61 | 0.4 |  | 1 | 24 |  | 6.47 | 0.02 |
| Mn | 1 | 13 |  | 0.14 | 0.7 |  | 1 | 24 |  | 4.41 | 0.05 |
| Na | 1 | 13 |  | 1.41 | 0.3 |  | 1 | 22 | * | 30.2 | < 0.0001 |
| Ni | 1 | 13 |  | 0.47 | 0.5 |  | 1 | 9 | * | 50.4 | < 0.0001 |
| P | 1 | 10 | * | 8.56 | 0.02 |  | 1 | 24 |  | 1.08 | 0.3 |
| S | 1 | 13 |  | 4.50 | 0.05 |  | 1 | 24 |  | 17.2 | 0.0004 |
| Si | 1 | 13 |  | 1.51 | 0.2 |  | 1 | 24 |  | 11.6 | 0.002 |
| Sr | 1 | 13 |  | 0.26 | 0.6 |  | 1 | 24 |  | 1.30 | 0.3 |
| Zn | 1 | 13 |  | 0.022 | 0.9 |  | 1 | 10 | * | 5.13 | 0.05 |
